# The Glucagon-Like Adipokinetic Hormone in *Drosophila melanogaster* – Biosynthesis and Secretion

**DOI:** 10.3389/fphys.2021.710652

**Published:** 2021-12-23

**Authors:** Bryon N. Hughson

**Affiliations:** Department of Ecology and Evolutionary Biology, University of Toronto, Toronto, ON, Canada

**Keywords:** adipokinetic hormone, corpora cardiaca, K_ATP_ channels, AMPK, PKG, metabolism, AKH, *Drosophila melanogaster*

## Abstract

Metabolic homeostasis requires the precise regulation of circulating sugar titers. In mammals, homeostatic control of circulating sugar titers requires the coordinated secretion and systemic activities of glucagon and insulin. Metabolic homeostasis is similarly regulated in *Drosophila melanogaster* through the glucagon-like adipokinetic hormone (AKH) and the *Drosophila* insulin-like peptides (DILPs). In flies and mammals, glucagon and AKH are biosynthesized in and secreted from specialized endocrine cells. K_ATP_ channels borne on these cells respond to fluctuations in circulating glucose titers and thereby regulate glucagon secretion. The influence of glucagon in the pathogenesis of type 2 diabetes mellitus is now recognized, and a crucial mechanism that regulates glucagon secretion was reported nearly a decade ago. Ongoing efforts to develop *D. melanogaster* models for metabolic syndrome must build upon this seminal work. These efforts make a critical review of AKH physiology timely. This review focuses on AKH biosynthesis and the regulation of glucose-responsive AKH secretion through changes in CC cell electrical activity. Future directions for AKH research in flies are discussed, including the development of models for hyperglucagonemia and epigenetic inheritance of acquired metabolic traits. Many avenues of AKH physiology remain to be explored and thus present great potential for improving the utility of *D. melanogaster* in metabolic research.

## Feeding Behavior, Metabolism and Homeostasis

All animals share a common unifying trait: the need to feed. The decision to search for and then ingest food must be balanced against other physiological considerations that contribute to an organism’s health and fitness. The regulation of physiological traits within a narrow range around precise setpoints is fundamental to the health and survival of an organism (Bernard, 1878). This process of maintaining the “milieu intérieur” around a steady state is homeostasis (Bernard, 1854; Cannon, 1929). Metabolic homeostasis comprises all processes that maintain a steady internal energy state. Fundamental to this process is the ability to detect and respond to perturbations in metabolic homeostasis through inter-organ communication of energy status (Rajan and Perrimon, 2011).

*Drosophila melanogaster* presents great potential for the study of metabolic homeostasis. In both humans and flies, perturbations in metabolic homeostasis are detected as changes in circulating sugar titers; specifically, blood glucose titers in humans, and hemolymph (i.e., the insect “blood”) glucose and trehalose titers in flies (Chown and Nicholson, 2004). Trehalose is a non-reducing disaccharide that functions as a store of glucose whose levels fluctuate broadly in the hemolymph (Chown and Nicholson, 2004). Metabolic homeostasis is regulated through the coordinated actions of peptide hormones that mediate autocrine, paracrine and endocrine signaling within/between organs (Droujinine and Perrimon, 2016). This coordination is achieved through cell autonomous nutrient-sensing of circulating glucose titers (Kim and Rulifson, 2004; Rorsman et al., 2014).

The mammalian pancreatic α and β cells are cell-autonomous nutrient sensors that monitor blood glucose homeostasis. The response to changes in blood glucose homeostasis must be rapid and efficient. Chronically reduced glucose (hypoglycemia) decreases energy production in metabolizing tissues and can cause death, and chronically increased glucose (hyperglycemia) produces reactive oxygen species that cause cytokine storm-induced apoptosis (Verzola et al., 2004; Brownlee, 2005; Cryer, 2007; Volpe et al., 2018).

α cells secrete glucagon in response to hypoglycemia and β cells secrete insulin in response to hyperglycemia (Rorsman et al., 2014). Fruit flies similarly use a glucagon-like peptide called the adipokinetic hormone (AKH) and eight *Drosophila* insulin-like peptides (DILP1–8) to regulate hemolymph (insect blood) glucose levels (Ikeya et al., 2002; Rulifson et al., 2002; Kim and Rulifson, 2004; Lee and Park, 2004; Alfa and Kim, 2016). AKH is biosynthesized in and secreted from the corpora cardiaca (CC), and DILP1, −2, −3, and −5 are biosynthesized in and secreted from the insulin-producing cells (IPCs) in the pars intercerebralis (PI) of the protocerebrum (Brogiolo et al., 2001; Ikeya et al., 2002; Rulifson et al., 2002; Kim and Rulifson, 2004; Lee and Park, 2004). Due to their α and β cell-like functions, the CC and IPCs are together viewed functionally as the fly pancreas. The CC is also known as the AKH producing cells (APCs)–this review will use the original CC nomenclature in referring to this tissue.

The catabolic actions of glucagon and anabolic actions of insulin are antagonistic, and the function of one in the homeostatic regulation of blood/haemolymph glucose titers must be studied in the context of the other (Unger and Orci, 1975). This relationship is more complex in *Drosophila*, where AKH and all eight DILPs do not always act in an antagonistic manner, nor do all DILPs regulate haemolymph glucose titers; this will be expanded upon below. Another important distinction between mammals and *Drosophila* is that–unlike glucagon–AKH does not catabolize glycogen, which is stored along with lipids in the fat body (Grönke et al., 2007; Kuhnlein, 2010; Gàlikovà et al., 2015). In flies, the *target of brain insulin* (*tobi*) is thought to convert glycogen stores to glucose; interestingly, *tobi* is regulated systemically by both AKH and DILP (perhaps DILP3) in response to protein and sugar intake, and ablation of either the IPCs or CC eliminates *tobi* expression and promotes glycogen accumulation (Buch et al., 2008).

Where specific DILPs do act similarly to mammalian insulin and regulate haemolymph glucose titers, the precise regulation of glucagon/AKH and insulin/DILP biosynthesis, secretion, ligand-receptor binding on target cells, and signal transduction within the target cell are central to homeostatic regulation of blood/haemolymph glucose titers. Pathogenesis of metabolic syndrome proceeds from the dysregulation of any of these four processes in glucagon/insulin physiology (Alfa and Kim, 2016). Metabolic research focused preferentially on insulin, and the regulation of glucagon/AKH physiology is not as well characterized (Lund et al., 2014; Alfa and Kim, 2016; Onyango, 2020). Growing insight into the contribution of glucagon to the pathogenesis of diabetes as well as further characterization of regulatory mechanisms for glucagon secretion invigorated this field of research (Leiss et al., 2011; Zhang et al., 2013; Gàlikovà et al., 2015; Palu et al., 2017; Song et al., 2017; Braco et al., 2020; Kellard et al., 2020). These developments make a review of this research timely.

An authoritative review of the catabolic action of AKH on the insect fat body is published elsewhere (Heier and Kühnlein, 2018). This review focuses primarily on the regulation of AKH activity through its biosynthesis and secretion from the CC, and covers seminal research performed using rodents. Current understanding–gleaned from murine and fly research–of the mechanism whereby CC cell autonomous nutrient sensing is mediated by ATP-sensitive potassium (K_ATP_) channels is reviewed in detail. The role that this mechanism plays in regulating insulin secretion must also be discussed, as the physiology of glucagon and insulin cannot be thoroughly investigated independently of one another (Unger and Orci, 1975). This review closes with a discussion of the application of CC and AKH research to the development of fly models for metabolic syndrome. Strengths and weaknesses of existing fly models are addressed.

## The Corpora Cardiaca and Adipokinetic Hormone

### Corpora Cardiaca Developmental Origins and Anatomy

At 25°C and in a nutrient abundant environment, embryogenesis in *D. melanogaster* requires 24 h and is followed–over 4 days–by three larval instars, wandering behavior, and pupariation. The larval body plan is reorganized during pupal metamorphosis and the sexually mature adult ecloses from its puparium 5 days after pupariation (Ashburner, 1989). The CC is a bilobed neuroendocrine tissue that develops from a pair of neuroblasts in the stage 10 embryo. It is unclear whether these cells originate from head mesoderm or from anterior neuroectoderm (de Valesco et al., 2004; Wang et al., 2007; Park et al., 2011). The neural stem cell progenitors of the CC were reported to originate from the anterior neuroectoderm because these cells expressed genes that are orthologous to those expressed in progenitors of the mammalian anterior pituitary gland (Wang et al., 2007). This suggests common evolutionary origins of neuroendocrine control of metabolism.

During larval life, the CC–along with the prothoracic gland (PG) and corpus allatum (CA)–forms part of the ring gland and is comprised of approximately seven cells per lobe in third instar larvae (Lee and Park, 2004). The ring gland is located dorsally on the central nervous system just anterior to the brain, and the gut and heart pass through the “ring” along the body midline. During pupal metamorphosis, the CC cells are conserved and migrate posteriorly to the brain lobes to sit on the dorsal surface of the foregut and anterior to the cardia. The adult CC cells are positioned around the heart and the CA sits on the dorsal surface of the CC; the PG is lost during metamorphosis. The distinction between lobes in the adult CC is not readily discernible, and the adult CC was reported to consist of 11–16 cells (Lee and Park, 2004).

Neural projections from the CC extend to the heart, esophagus and central nervous system in adults and larvae, as well as the larval PG and the adult crop (Kim and Rulifson, 2004; Lee and Park, 2004). Recent work has identified the IPCs as targets of CC axonal projections to the larval CNS where they regulate the sugar-dependent secretion of DILP3 (Kim and Neufeld, 2015). The IPCs also target the CC and DILP2–whose secretion is stimulated by amino acids–was identified in a subset of CC cells (Rulifson et al., 2002; Géminard et al., 2009). CC axonal projections to the heart transport endocrine peptides to the hemolymph for dispersal throughout the body. AKH secreted from these neurons also stimulates heart contractions to improve dispersal to target tissues (Noyes et al., 1995). IPC neural projections also extend to the heart where they have extensive contact with CC axons (Kim and Rulifson, 2004). The function–if any–of these connections is unknown.

A single pair of neurons extends from the adult CC to the crop and has been hypothesized to regulate crop emptying in *D. melanogaster* (Lee and Park, 2004). The crop is the primary storage organ for carbohydrates in flies, as muscle and fat body glycogen stores are very small (Chown and Nicholson, 2004). The regulation of crop emptying is crucial to the early response to starvation. In the blowfly, *Phormia regina*, these neurons extend down the crop duct where they project onto the supercontractile muscles sheathing the crop (Stoffolano et al., 2014). AKH is transported to these muscles and stimulates muscle contraction and crop emptying.

Corpora cardiaca axons containing AKH project to the PG: this was reported in *D. melanogaster*, but their function is unknown (Kim and Rulifson, 2004). The PG is the site of biosynthesis and secretion of ecdysteroids and plays a crucial role in regulating the timing of developmental transitions during larval life (Yamanaka et al., 2013). This CC neuroanatomy prompted the hypothesis that the CC and AKH may influence development through the regulation of ecdysteroid physiology. This hypothesis is interesting in light of the role of the PG as a “decision-making center” that alters developmental timing according to changes in energy homeostasis (Yamanaka et al., 2013). However, CC ablation, AKH receptor (AKHR) mutant, and AKH mutant experiments did not identify a developmental role for the CC or AKH (Kim and Rulifson, 2004; Lee and Park, 2004; Grönke et al., 2007; Bharucha et al., 2008; Gàlikovà et al., 2015). Recent work identified a nutrient-dependent role for AKH in larval development, as well as its influence on the PG; this is discussed further below (Hughson et al., 2021).

### The Adipokinetic Hormone Gene and Peptides

The *D. melanogaster akh* gene (also: *dAkh*; hereafter referred to as *akh*) was sequenced and localized between 64A10 and 64B1, 2 on chromosome 3L (Noyes et al., 1995). Consisting of 2 exons, the first of which encodes the signal sequence and first amino acid residue (pGlu, pyroglutamic acid), *akh* encodes a polyadenylated mRNA approximately 600 bases in length. Noyes et al. (1995) noted that although both the pre-propeptide and active AKH peptide of *D. melanogaster* display high conservation in structure with the AKH of other insect species, the intron-exon sequences and C terminal peptide sequences do not show this same high degree of conservation. *akh* mRNA expression was localized exclusively to the larval and adult CC and all or nearly all CC cells express *akh* (Noyes et al., 1995; Kim and Rulifson, 2004; Lee and Park, 2004).

The *D. melanogaster* AKH peptide (DAKH) (hereafter referred to as AKH) is biosynthesized in and secreted from the CC cells. AKH is synthesized as a pre-prohormone that is processed to its bioactive form by the pre-prohormone convertase *amontillado* (*amon*) (Rhea et al., 2010). The DAKH is an octomer (pGlu-Leu-Thr-Phe-Ser-Pro-Asp-Trp-NH_2_) (Schaffer et al., 1990).

The identification of AKH peptides in other insect species revealed that although conservation of gene and peptide structure was evident, variation in amino acid sequence at the seventh position imparts significant consequences for AKH bioactivity. The seventh amino acid is aspartic acid in *D. melanogaster* and is asparagine in other insects (Schaffer et al., 1990). *In vivo* assays reported a 100-fold reduction in the activity of *D. melanogaster* AKH in the grasshopper *Schistocerca nitans* whose AKH bears asparagine at this seventh position. Aspartic acid is thought to impart a charge on AKH at physiological pH that is absent in AKH bearing asparagine. The charged *D. melanogaster* AKH is hypothesized to produce a poor interaction with receptors for uncharged AKH peptides. The AKHR will be discussed below.

As with *Akh* mRNA expression, AKH-immunoreactivity (IR) patterns were restricted to the CC in whole mount immunohistochemical analyses of third instar larval and adult tissues using antibodies against AKH (Kim and Rulifson, 2004; Lee and Park, 2004; Isabel et al., 2005). Third instar larvae contain between 110 and 120 fmol AKH/fly, and do not show sex differences in whole body AKH levels (Noyes et al., 1995). Whole body AKH levels in adult flies are not strain dependent (i.e., Canton S vs. Oregon R), but AKH peptide content in 7–9 d.o. females is greater than in males (average: 139 ± 45 fmol/female vs. 103 ± 33 fmol/male) (Noyes et al., 1995). However, within sexes, no correlation exists between adult body weight and whole body AKH peptide content (Noyes et al., 1995).

The biosynthesis of endocrine compounds is not necessarily coupled with secretion (Harthoorn et al., 2001). This is evident in AKH physiology where *akh* transcriptional activity and pre-prohormone processing are not affected by secretory stimuli (Harthoorn et al., 2001; Diederen et al., 2002; Lee and Park, 2004). Further evidence that AKH biosynthesis is tightly regulated comes from the observation that flies with 1 or 3 copies of *akh* all contain similar levels of AKH (Noyes et al., 1995). These data suggest that at all times AKH biosynthesis proceeds in the CC at such a rate that sufficient AKH protein is stored within secretory granules to meet the animal’s fluctuating physiological needs.

Bioactive AKH is processed from a pre-propeptide that also yields the AKH precursor-related peptide (APRP) (Hekimi and O’Shea, 1987). APRP is functionally orphan and does not affect carbohydrate or lipid metabolism in *Locusta migratoria* and the grasshopper *Romalea microptera* (Oudejans et al., 1991; Hatle and Spring, 1999). APRP belongs to the growth hormone-releasing factor (GRF) superfamily, which includes glucagon, glucagon-like peptides 1 and 2, and the GRF peptide itself. APRP shares the greatest peptide sequence homology with the mammalian GRF (De Loof and Schoofs, 1990; Clynen et al., 2004). GRF influences developmental progression in mammals, and this prompted the hypothesis that APRP is functionally homologous to GRF–that is, APRP is a putative developmental regulator. However, APRP was demonstrated to have neither ecdysiotropic effects in *L. migratoria*, nor to influence the ecdysteroid-dependent timing of developmental transitions in *D. melanogaster* (De Loof et al., 2009; Gàlikovà et al., 2015).

### Regulatory Inputs to the Corpora Cardiaca

The CC receives both extrinsic and intrinsic regulatory inputs. An AKH-mediated autocrine feedback loop negatively regulates *akh* expression (Gàlikovà et al., 2015). Nutrition directly influences CC glucose metabolism in a manner that regulates cell membrane electrical activity; this is discussed thoroughly in the next section of this review. Other nutrient-derived factors include: the muscle-derived cytokine unpaired-2 (Upd2) that alters AKH secretion (Zhao and Karpac, 2017); α-bursicon is secreted from enteroendocrine cells and signals through Dlgr2 to negatively regulate AKH production and systemic signaling (Scopelitti et al., 2019); epistatic interaction between DILP1 and −2 regulate *akh* expression and intracellular AKH abundance (Post et al., 2019); gut- and brain-derived allatostatin-A (AstA) targets the CC and IPCs to positively regulate AKH and negatively regulate DILP (Hentze et al., 2015); pigment-dispersing factor (PDF) and dopamine promote intracellular Ca^2+^ and increase locomotion during starvation (Braco et al., 2020). Additionally, the gustatory water-sensing ion channel *pickpocket28* (*ppk28*) negatively regulates AKH secretion (Waterson et al., 2010). These and other factors that provide regulatory input to the CC are reviewed elsewhere (Nässel and Winther, 2010; Ahmad et al., 2019). Mechanisms and anatomy of neural regulatory inputs to the CC–such as α-bursicon/Dlgr2, AstA, and sNPF–are authoritatively reviewed by Nässel and Zandawala (2020).

### The Adipokinetic Hormone Receptor

The physiological effects of AKH are mediated by the *D. melanogaster* AKH receptor (DAKHR; also AKHR). *D. melanogaster AkhR* is the first insect *AkhR* gene reported to encode a seven-transmembrane domain G-protein coupled receptor, or GPCR (Staubli et al., 2002). AKHR is structurally- and evolutionarily-related to the mammalian gonadotropin-releasing hormone (GnRH) receptor, and was named originally named GnRHR (Staubli et al., 2002; Zandawala et al., 2018). When the ligand of the *D. melanogaster* GnRHR was identified as AKH, this receptor was renamed AKHR (Staubli et al., 2002). AKHR will be discussed further below.

*In situ* localization of *AkhR* expression and AKHR protein abundance within the fly is complicated by the difficulty in distinguishing between highly conserved regions of GPCRs. Reporter gene expression driven by *AkhR*-GAL4 in adults suggests that *AkhR* is expressed in the fat body tissues of the head and abdomen (Bharucha et al., 2008). *AkhR*-GAL4 also drives expression in the sweet sensing gustatory receptor neurons of the suboesophageal ganglion (SOG) (Bharucha et al., 2008). These *AkhR*-GAL4-driven expression patterns are not comprehensive. Tissue-specific expression of *AkhR*-RNAi also identified roles for AKHR signaling in the IPCs, the PG, and in four interoceptive SOG neurons (ISNs) (Kim and Neufeld, 2015; Jourjine et al., 2016; Hughson et al., 2021).

## *Drosophila* Insulin-Like Peptide Physiology

Although this review is AKH-centric, it must include some discussion of DILPs because AKH secretion must be coordinated with insulin/insulin-like growth factor signaling (IIS) where haemolymph glucose homeostasis is concerned (Under and Orci, 1975; Owusu-Ansah and Perrimon, 2014). Eight DILPs are produced in *Drosophila* and their expression patterns and functions vary according to discrete stages of life; this information is thoroughly reviewed elsewhere (Owusu-Ansah and Perrimon, 2014; Nässel and Vanden Broeck, 2016). The regulation of DILP biosynthesis, secretion, and signaling in *Drosophila* is very complex, and only a brief summary can be provided here.

Prior to adult life, DILP2, −3, and −5 are produced in the IPCs and embryonic tissues, as well as the larval imaginal disks (DILP2); these three DILPs regulate growth, development, and metabolic homeostasis through the IIS pathway; DILP1 is also produced in the IPCs and affects growth. During adult life DILP2, −3, and −5 are produced in the IPCs and midgut (DILP3), and affect metabolic homeostasis, fecundity, stress resistance, and lifespan. Secretion of DILP2, −3, and −5 is regulated by nutrients (carbohydrates, lipids, and proteins), and neuroendocrine factors, including AKH, serotonin, octopamine, and fat body-derived Upd2 and DILP6. The other four DILPs are produced in the embryo, larval gut, fat body, neuroblasts, imaginal disks, and nervous system where they regulate life stage- and nutrient-specific growth (DILP1, −4, −6), reproductive decisions (DILP7), and development (DILP6, −8).

Insulin signaling in *Drosophila* is more complex than in mammals, and it is inappropriate to generalize all DILPs as being direct actors in metabolic homeostasis in the same sense that β cell-derived insulin is in mammals. It is similarly inappropriate to oversimplify the activities of AKH and all DILPs as being antagonistic. However, specific DILPs do directly alter haemolymph glucose titers (e.g., DILP2, −3), and this review refers to the activities of AKH and these DILPs as being antagonistic.

## Coordination of Adipokinetic Hormone/Glucagon and *Drosophila* Insulin-Like Peptide/Insulin Secretion

Adipokinetic hormone and DILP secretion must be coordinated so that their antagonistic actions preserve hemolymph glucose homeostasis. Central to this goal is the ability of the CC and IPCs to monitor energy homeostasis through cell autonomous nutrient sensing of circulating glucose titers. This is accomplished by the evolutionarily conserved K_ATP_ channels. Most of the research that characterized the K_ATP_ channel-regulated secretion of glucagon/AKH and insulin/DILPs was conducted in mice and the focus of this portion of the review will begin with mammalian α and β cells before returning to *D. melanogaster*.

### ATP-Sensitive Potassium Channels Confer Cell Autonomous Nutrient Sensing

ATP-Sensitive Potassium channels function as cell autonomous nutrient sensors in mammalian pancreatic α and β cells (for comprehensive reviews, see: Ashcroft and Rorsman, 2013; Rorsman et al., 2014), and in the *D. melanogaster* larval and adult CC; these channels are also present in the adult–but not larval–IPCs (Kim and Rulifson, 2004; Fridell et al., 2009; Kréneisz et al., 2010). K_ATP_ channels also contribute to healthy mammalian and *Drosophila* cardiac function (Akasaka et al., 2006). The K_ATP_ channel contains two subunits comprised of four regulatory sulfonylurea receptors (SURx: SUR1 in mammals; *Sur* in flies) and four pore-forming weakly inward rectifying potassium channels (Kir6.x: Kir6.2 in mammals; *KirI*-*III* in flies) (Clement et al., 1997; Nasonkin et al., 1999; Döring et al., 2002; Ashcroft and Rorsman, 2013). Kir6.x bears an ATP-binding domain and SURx bears a Mg-ADP-binding domain: Kir6.x-ATP binding stimulates channel closure and cell membrane depolarization; SURx-Mg-ADP stimulates channel opening and cell membrane polarization.

The nutrient sensing capacity of the K_ATP_ channel is conferred by the sensitivity of its two subunits to changes in intracellular adenosine triphosphate/magnesium-adenosine diphosphate (ATP/Mg-ADP) ratio. The movement of K^+^ ions out of the cell through open K_ATP_ channels maintains a polarized resting membrane potential of −70 mV. The weakly inward rectifying action of the Kir6.x subunit comes into play upon K_ATP_ channel closure: inward flow of K^+^ ions now predominates over K^+^ efflux and the cell membrane depolarizes (Ashcroft and Gribble, 1999; Hibino et al., 2010).

#### Glucose Metabolism Alters the ATP/Mg-ADP Ratio

Glucose transporters bring glucose into the mouse α (GLUT1 and GLUT4) and β (GLUT2) cells where it enters the Krebs cycle for ATP production (Heimberg et al., 1995; Bähr et al., 2012). In hyperglycemic conditions, elevated glucose metabolism increases the ATP/Mg-ADP ratio, Kir-ATP binding predominates over SUR-Mg-ADP, and K_ATP_ channels close (Ashcroft and Rorsman, 2013). This stimulates cell membrane depolarization and–along with an unidentified depolarizing current–causes action potential firing that regulates insulin and glucagon secretion (Rorsman et al., 2014). Paradoxically, K_ATP_ channel closure causes depolarization and action potential firing in both cell types but promotes insulin secretion from β cells while inhibiting glucagon secretion from α cells. The means whereby α and β cell K_ATP_ channels produce opposite effects on secretion in response to the same glucose titers is still incompletely understood. Recent work demonstrated that this is likely due to differential glucose sensitivity of K_ATP_ channels between the two cell types, which significantly alters cellular excitability and action potential firing (Göpel et al., 2000; Zhang et al., 2013). I will review electrical regulation of both α and β cells in order to contrast important differences in effect of K_ATP_ channel activity between the two cell types.

#### Ion Channels Regulate Cell Electrical Activity and Secretion

Insulin secretion from β cells is stimulated by cell membrane depolarization caused by K_ATP_ channel closure (Cook and Hales, 1984; Rorsman et al., 2012). β cell K_ATP_ channel conductance is high (i.e., many channels are open) at 1 mM glucose, and cell membrane depolarization, action potential firing, and insulin secretion are inhibited (Zhang et al., 2013). When glucose concentrations rise above 6 mM, the intracellular ATP/Mg-ADP ratio increases and induces K_ATP_ channel closure. Subsequent cell membrane depolarization and action potential firing activate L-type voltage-gated calcium channels (VGCC) and increase intracellular calcium [Ca^2+^]_*i*_. Ca^2+^ accumulation in the immediate area near the VGCCs promotes Ca^2+^-mediated exocytosis of insulin-containing granules. Electrical regulation of insulin secretion from β cells is thoroughly reviewed elsewhere (Sarmiento et al., 2019; Zhang et al., 2020).

α cells are more sensitive to glucose than are β cells. In contrast to β cells, α cells show very low K_ATP_ channel conductance at 1 mM of glucose; this results from α cell K_ATP_ channels having a 5-fold greater sensitivity to ATP produced by glucose phosphorylation (Zhang et al., 2013). As a result, small changes in K_ATP_ channel activity (i.e., closure of a small number of highly sensitive K_ATP_ channels in α cells) at low glucose concentrations produce large, rapid effects on action potential generation and firing (Barg et al., 2000; Leung et al., 2006; Zhang et al., 2013). The high sensitivity of these K_ATP_ channels is crucial as it makes α cells electrically active at low glucose concentrations, thus stimulating VGCC activity and permitting glucagon secretion at glucose concentrations that inhibit insulin secretion from β cells. The crucial role played by voltage-gated sodium channels (VGSCs) in mediating this process in α cells that is described below.

Just as importantly–because glucagon must be secreted only during hypoglycemia–increasing glucose concentrations to 6 mM rapidly closes all remaining α cell K_ATP_ channels. This produces strong membrane depolarization and action potential firing, and–through the activity of VGSCs–prevents glucagon secretion during hyperglycemia. Glucagon secretion is thus both stimulated and inhibited by varying magnitudes of K_ATP_ channel activation. This relation between K_ATP_ channel conductance and glucagon secretion follows an inverted U-shaped dose response curve where maximum secretion occurs at 1 mM glucose, and small increases or decreases in conductance inhibit secretion (Zhang et al., 2013).

α cell membranes bear VGSCs that are activated by K_ATP_ channel closure and membrane depolarization at 1 mM glucose. VGSC activation is essential for glucagon secretion because Na^+^ influx increases the action potential spike height necessary for activating VGCCs and promoting Ca^2+^ entry at low glucose concentrations (Barg et al., 2000; Zhang et al., 2013). The VGSCs produce rapid and short-lived amplification of the moderate depolarizing stimulus that is produced by K_ATP_ channel closure at low glucose concentrations. When all α cell K_ATP_ channels close in response to hyperglycemia, further membrane depolarization increases action potential firing and inactivates VGSCs. VGSC closure reduces action potential firing and spike height, VGCCs are subsequently inactivated, and glucagon secretion is inhibited. Voltage-gated potassium channels open slowly during cell membrane depolarization to permit the repolarizing K^+^ efflux that is required for reactivation of VGSCs and continuation of glucagon secretion (Spigelman et al., 2010).

In contrast to β cells, three types of VGCCs–L-type, P/Q-type, and T-type–are present in mouse α cells (Barg et al., 2000; Leung et al., 2006; Zhang et al., 2013). This combination of Ca^2+^ channel types appears to optimize accumulation of [Ca^2+^]_*i*_ at low glucose concentrations and initiation of action potential firing. The majority of the Ca^2+^ current passes through L-type channels but P/Q channels exert strong influence over glucagon secretion (Zhang et al., 2013; Rorsman et al., 2014). This is because the activity of P/Q-type channels depends upon the increased spike amplitude produced by VGSC activity, which is inhibited by strong membrane depolarization that occurs at high glucose concentrations. As in β cells, [Ca^2+^]_*i*_ increases in the α cell sub-membrane locale of VGCCs and promotes glucagon secretion.

The precise regulation of ion channel activity through K_ATP_ channel-mediated nutrient sensing underlies the proper timing of glucagon and insulin secretion. Homeostatic control of blood glucose homeostasis is highly sensitive to any factors that dysregulate channel function. Mutations in K_ATP_ channels, VGCCs, and VGSCs that perturb their function are currently the focus of research aimed at identifying causal mechanisms that underlie the pathogenesis of diabetes.

The precise mechanism whereby α cell membrane electrical activity and glucagon secretion are regulated remains to be elucidated. Although glucose-dependent K_ATP_ channel activity certainly plays a prominent role in regulating the inverted U-shaped curve of α cell membrane electrical activity described above, it is not the sole determinant of glucagon secretion. Evidence suggests that K_ATP_ channel-independent mechanisms can contribute to this pattern of electrical activity and produce glucagonostatic effects at high glucose concentrations through both extrinsic (paracrine) and intrinsic means. These include the regulation of α cell electrical activity by insulin and somatostatin (secreted from δ islet cells), a Ca^2+^ store operated current (SOC), and direct inhibition of P/Q type VGCCs by a glucose metabolite (Hauge-Evans et al., 2009; De Marinis et al., 2010; Unger and Orci, 2010; Watts et al., 2016; Vergari et al., 2019; Zhang et al., 2020).

### Protein Kinases Regulate [Ca^2+^]_*i*_

In mouse α and β cells, the adenosine monophosphate-activated protein kinase (AMPK) and the cGMP-dependent protein kinase (PKG) regulate insulin and glucagon secretion in response to changes in K_ATP_ channel activity and [Ca^2+^]_*i*_, but serve in reverse roles between cell types as positive and negative regulators of secretion (Rorsman et al., 2014). In β cells, insulin secretion is inhibited by AMPK and stimulated by PKG (Granot et al., 2009; Undank et al., 2017); in α cells, glucagon secretion is stimulated by AMPK and inhibited by PKG (Leclerc et al., 2011; Leiss et al., 2011). Both protein kinases exert their regulatory effects through both modulation of and in response to changes in K_ATP_ channel activity and [Ca^2+^]_*i*_ levels.

#### AMP-Activated Protein Kinase and Protein Kinase in β Cells

Insulin secretion is inhibited by hypoglycemia and decreased ATP production in β cells–this promotes K_ATP_ channel conductance, the cell membrane hyperpolarizes, and [Ca^2+^]_*i*_ decreases. This is mediated by mechanisms that are both dependent and independent of AMPK. Liver kinase B1 (LKB1) induces AMPK activity to inhibit insulin biosynthesis and secretion in both a glucose- and amino acid-responsive manner (da Silva Xavier et al., 2003; Leclerc and Rutter, 2004; Granot et al., 2009; Shackelford and Shaw, 2009). AMPK also inhibits insulin secretion via a leptin-mediated feedback loop (Tsubai et al., 2016). In response to feeding, insulin promotes leptin secretion from adipose tissue, and leptin signaling in β cells subsequently induces protein kinase A (PKA) activation of AMPK (Park et al., 2013; Cochrane et al., 2020). Leptin-PKA-AMPK signaling regulates membrane polarity–and thus cell excitability–by promoting K_ATP_ channel trafficking to the β cell membrane (Cochrane et al., 2020). This increases K_ATP_ channel conductance and hyperpolarizes the cell membrane. Importantly, this occurs only in a progressively fasted state when the glucose:leptin titer ratio decreases to a level where continued insulin secretion would produce a hypoglycemic state (Park et al., 2013). Greatly elevated [Ca^2+^]_*i*_–induced by hyperglycemia and glucose-stimulated ATP production–also promotes AMPK activity to inhibit insulin secretion; this seems contradictory given that hyperglycemia promotes insulin secretion, and it was postulated to function as a self-regulatory feedback mechanism that prevents excessive insulin secretion and hypoglycemia (Leclerc and Rutter, 2004; Granot et al., 2009).

In β cells, PKG promotes insulin secretion in a fed state either by phosphorylating and closing K_ATP_ channels or by phosphorylation of proteins that indirectly target K_ATP_ channels (Soria et al., 2004; Undank et al., 2017). PKG activity is induced by atrial natriuretic peptide (ANP) signaling in β cells (Undank et al., 2017). PKA also phosphorylates and closes K_ATP_ channels, and PKG promotes this inhibitory effect by preventing phosphodiesterase deactivation of PKA (Undank et al., 2017). This PKA-mediated increase in insulin secretion appears contradictory to its inhibitory effect reported above; however, PKA inhibition of K_ATP_ channels is mediated by PKG signaling, whereas leptin-PKA-AMPK signaling increases K_ATP_ channel conductance in the absence of PKG activity (Undank et al., 2017; Cochrane et al., 2020). PKG was also reported to inhibit insulin secretion by directly phosphorylating L-type VDCCs and decreasing [Ca^2+^]_*i*_; as with the effect of elevated [Ca^2+^]_*i*_ on AMPK, this might serve as a self-regulatory feedback loop (Sandoval et al., 2017). The complexity of the glucose-responsive and self-regulatory pathways present in β cells reflects the need for rapid responses in insulin secretion to changes in blood glucose levels.

#### AMP-Activated Protein Kinase and Protein Kinase in α Cells

In α cells, AMPK activity is required for increased [Ca^2+^]_*i*_ following the moderate depolarizing stimulus produced by the opening of a small number of K_ATP_ channels (Leclerc et al., 2011; Zhang et al., 2013). AMPK activation is induced by AMP following a decrease in the ATP/AMP ratio in hypoglycemic conditions. This promotes glucagon secretion through an incompletely characterized signaling cascade (Leclerc et al., 2011; Onyango, 2020). Subsequent to [Ca^2+^]_*i*_ increase, AMPK is thought to stimulate glucagon secretion either by increasing the abundance or the fusion competence of glucagon-containing secretory granules at the cell membrane (Leclerc et al., 2011). Induction of AMPK by AMP is mediated by LKB1 during hypoglycemia, but AMPK is not the sole target of LKB1 phosphorylation in glucagon regulation (Sun et al., 2015).

In α cells, PKG responds to closure of all K_ATP_ channels by inhibiting an increase in [Ca^2+^]_*i*_ (Leiss et al., 2011). Unlike in β cells, the PKG phosphorylation target(s) is unknown, but its activity inhibits the VGCC-mediated increases in [Ca^2+^]_*i*_ that are required for glucagon secretion at low glucose concentrations (Ropero et al., 2002; Zhang et al., 2013). This physiological effect suggests that–as in β cells–PKG might phosphorylate VGCCs and close these channels. Further insight is provided by research into cardiac myocytes where nitric oxide stimulated PKG activity inhibits Ca_*v*_1.2 VGCCs directly phosphorylating the channel (Yang et al., 2007).

### ATP-Sensitive Potassium Channels, AMP-Activated Protein Kinase, Protein Kinase, and Adipokinetic Hormone Secretion in *D. melanogaster*

The murine research reviewed above informs future research into the regulatory mechanisms of AKH secretion in *D. melanogaster*. Endocrine research requires the ability to quantify changes in hormone secretion. However, circulating AKH titers in *D. melanogaster* are estimated to be in the low femtomolar range, and this makes the reliable quantification of AKH titers an ongoing challenge that will be addressed below (Isabel et al., 2005; Ahmad et al., 2019).

Pharmacological and transgenic manipulations were used to implicate K_ATP_ channels in the regulation of AKH secretion from the larval CC (Kim and Rulifson, 2004). Tolbutamide is a diabetic drug that targets the Sur subunits of K_ATP_ channels. Larvae fed with tolbutamide showed a 40% increase in hemolymph glucose. Tolbutamide treatment was used in conjunction with transgenic manipulations where the CC was ablated to show that the increase in glucose titers was dependent upon the CC. The effect of tolbutamide was inhibited when CC membrane depolarization was transgenically inhibited. These experiments provided strong evidence for the existence of K_ATP_ channels in the CC and for their regulatory role in AKH secretion (Kim and Rulifson, 2004). Adult IPCs bear K_ATP_ channels, and *in vivo* electrophysiological measurements of these cells were used to discern the influence of K_ATP_ channels on membrane potential; the potential for applying this technique to the CC has not been explored (Fridell et al., 2009).

*In vivo* changes in CC [Ca^2+^]_*i*_ provided insight into the role of K_ATP_ channels and protein kinases in the regulation of AKH secretion. In dissected CC, the fluorescent Ca^2+^ indicator Camgaroo demonstrated that hypoglycemia increased [Ca^2+^]_*i*_ and hyperglycemia decreased [Ca^2+^]_*i*_ in response to hyperglycemia (Kim and Rulifson, 2004). CC [Ca^2+^]_*i*_ also increased in response to low trehalose levels, suggesting that the activity of trehalase borne on CC cell membranes might also serve as a nutrient sensor. These experiments were performed with tolbutamide treatment to infer the involvement of K_ATP_ channels in these [Ca^2+^]_*i*_ changes. These results supported a role for Ca^2+^-mediated vesicle exocytosis in AKH secretion (Kim and Rulifson, 2004).

A major contribution to the characterization of mechanisms that regulate CC cell membrane potential and AKH secretion was recently reported (Perry et al., 2020). Three genes that encode components of K_ATP_ channels (*Sur*), calcium channels (*Ca-Beta*), and potassium channels (*sei*) were identified through RNAi-mediated knockdown as regulatory candidates for excitation-secretion coupling for AKH in the CC. In total, 39 genes encoding components of K^+^, Ca^2+^, Na^+^, and Cl^–^ ion channels were expressed in the CC. These results provided further support for the nutrient-sensing role of K_ATP_ channels in the CC. This work also emphasized the importance of K^+^ efflux and Ca^2+^ influx in cell membrane depolarization and AKH secretion. The identification of CC ion channel components greatly improves the utility of *D. melanogaster* as a model for α cell dysregulation, hyperglucagonemia, and the pathogenesis of T2DM.

Further use of transgenic calcium indicators in the CC identified AMPK as a positive regulator of [Ca^2+^]_*i*_ and AKH secretion (Braco et al., 2012). CC [Ca^2+^]_*i*_ was influenced by changes in circulating glucose and trehalose titers as reported previously (Kim and Rulifson, 2004). Loss of AMPK function reduced secretion from the CC and diminished the increase in [Ca^2+^]_*i*_ that was induced by low trehalose (Braco et al., 2012). Additionally, induced AMPK activity was sufficient to increase [Ca^2+^]_*i*_ in a high trehalose environment.

The murine cGKI research described above prompted the hypothesis that PKG–encoded by *dg2* in *D. melanogaster*–might negatively regulate AKH secretion. Reduced *dg2* expression in the larval CC reduced intracellular AKH abundance, and this correlated with a low nutrient-dependent developmental delay and increased lethality prior to pupariation (Hughson et al., 2021). Compared to control genotypes, more of these larvae survived pupal metamorphosis and developed into adults with greater starvation resistance and increased body size to lipid content ratio, a trait associated with obesity in humans. This suggested that *dg2* functioned in the CC to increase survival during larval development in a low nutrient environment, and but that this resulted in a tradeoff with starvation resistance during adult life (Hughson et al., 2021). Further research demonstrated that *dg2* also influenced AKH abundance in the adult CC (Hughson, in press). Reduced *dg2* expression in the adult CC decreased intracellular AKH, but–in contrast to larvae–correlated with decreased body size to lipid content ratio. This effect correlated with evidence of increased systemic lipid catabolism and reduced starvation resistance during adult life.

## Evolutionary Conservation of Function

As described above, the CC is developmentally orthologous to the mammalian anterior pituitary gland (Wang et al., 2007). The PI (location of IPCs) of the fly protocerebrum is orthologous to the mammalian hypothalamus (Wang et al., 2007). Together, the hypothalamus and pituitary gland form the hypothalamus-pituitary (HP) “energy axis,” so named for its role in directing energy use throughout life. The functional conservation of the CC and IPCs as the fly “pancreas” in conjunction with the developmental orthology of these cells with the mammalian HP axis emphasizes the merit of studying CC-IPC function to the study of metabolic homeostasis. Although there is little conservation between AKH and glucagon amino acid sequences, both hormones act through the same evolutionarily conserved signaling pathway to regulate transcriptional responses to hypoglycemia (De Loof and Schoofs, 1990; Clynen et al., 2004; Song et al., 2017).

The HP axis also regulates the time of onset of puberty. When the HP axis detects a minimum level of body growth during childhood it stimulates steroid hormone biosynthesis in the gonads (Shalitin and Philip, 2003). The subsequent rise in steroid titers initiates the developmental transition from sexual immaturity to maturity. Paracrine signaling between the hypothalamus and pituitary gland is mediated by GnRH, which stimulates the secretion of gonadotropins that enter circulation and stimulate steroid hormone biosynthesis and secretion from the gonads. In *D. melanogaster*, steroid hormones similarly regulate the timing of this developmental transition.

The evolutionary relatedness of GnRHR and AKHR was introduced above. The conservation of AKHR and GnRHR prompted the hypotheses that AKHR influenced development by regulating ecydsteroidogenesis, and that AKH–in addition to its glucagon-like properties–possessed dual functionality as both a glucagon-like and GnRH-like peptide. Conserved peptide sequences between AKH and GnRH seemed to provide support for this hypothesis (Lindemans et al., 2009; Zandawala et al., 2018). However, recent work investigating AKH and AKHR loss of function mutant lines demonstrated that neither AKH nor AKHR affected developmental (i.e., hatchability, viability, time from egg laying to eclosion) or fitness (i.e., body size, fecundity) traits–neither AKH nor AKHR signaling are essential for survival (Gàlikovà et al., 2015).

While development was not altered in AKH loss of function mutants, recent work identified the possibility that AKH might play a role in ecdysteroid biosynthesis in the PG. Evidence comes from work demonstrating a role for AKH-regulated hormone sensitive lipase (HSL) activity in steryl ester metabolism and the intergenerational transfer of sterols (Heier et al., 2021). This pathway regulates catabolism of steryl ester lipid droplet stores and plays an essential role in ecdysteroid biosynthesis. While this work reported no effect of an HSL loss of function mutation on PG lipid droplets, these data came from animals reared in a lipid- and sterol-abundant feeding environment and larval development was not reported. The possibility that this pathway influences ecdysteroid biosynthesis in sterol-limited or -deficient environments needs to be explored.

This avenue of research is supported by a developmental role for AKH that was observed only in low nutrient conditions (Hughson et al., 2021). Larvae reared in a low nutrient (i.e., 75% reduction in carbohydrate, lipid, sterol, and protein content) feeding environment were developmentally delayed in response to a CC-specific manipulation of a PKG-encoding gene. This gene, *dg2*, is orthologous to cGKI, which encodes the PKG that regulates alpha cell membrane excitability (Leiss et al., 2011). This delay was AKH-dependent, and–as observed in AKH mutants reared in nutrient-abundant conditions–was absent in nutrient-abundant conditions (Gàlikovà et al., 2015). While these developmental effects were not linked to changes in glandular or circulating ecdysteroid titers, AKH significantly altered PG [Ca^2+^]_*i*_ levels (Hughson et al., 2021). This trait was associated with GPCR-mediated active secretion of ecdysteroids from the PG as well as with AKH activation of the HSL pathway (Yamanaka et al., 2015; Heier et al., 2021).

It is vital to reemphasize that AKH mutants did not exhibit developmental defects, delays, or fitness consequences, and that this definitively demonstrated that AKH is not essential for development in a nutrient-abundant environment (Gàlikovà et al., 2015). There is no contradiction between this seminal work and the report of an AKH-dependent effect on developmental timing that was present only in low nutrient conditions (Hughson et al., 2021). Instead, this identifies the possibility that in challenging nutritional environments AKH can play a non-essential role in development in a manner fitting for a stress peptide (Vogt, 1946). This hypothesis should be investigated in the context of nutrient abundance and stress over different developmental ages.

## The Future of Adipokinetic Hormone Research

The mechanisms that regulate AKH secretion must be characterized in order to improve the utility of *D. melanogaster* in metabolic research. Its small size puts the fly model at a disadvantage to rodent models in some respects; for example, electrophysiological assays performed using dissected and cultured α and β cells are rarely used in fly research (Fridell et al., 2009). However, flies possess traits that present an advantage over rodent models, such as a short life cycle and ease of controlling genetic background. One of the great strengths of *D. melanogaster* research is the ever-expanding library of transgenic lines that permit spatiotemporal-specific manipulations of CC function and AKHR signaling pathways.

This section discusses bioassays that can be established–or adapted from existing protocols–to improve fly models of metabolic syndrome. Some exciting avenues for future AKH research are also highlighted. First, a crucial weakness in *D. melanogaster* metabolic research must be addressed–the ability to quantify hemolymph sugar and AKH titers.

### Requirements for *D. melanogaster* in Metabolic Research

#### Circulating Metabolite Quantification

Dysregulation of blood glucose levels is diagnostic of pre-diabetic and diabetic states, and this phenotype is quantifiable in *D. melanogaster* metabolism research. Circulating AKH titers in *D. melanogaster* are estimated to be in the low femtomolar range and this makes the reliable quantification of AKH titers an ongoing challenge (Isabel et al., 2005; Ahmad et al., 2019). Unlike DILPs that are large enough to be tagged for quantification of secretion, the AKH octomer is too small for this technique (Park et al., 2014). This problem was circumvented by quantifying phenotypes that are predicted to indicate changes in AKH secretion. These surrogate methods include altered lifespan during starvation (Braco et al., 2012; Gàlikovà et al., 2015; Perry et al., 2020; Hughson et al., 2021; Hughson, in press), *ex vivo* immunohistochemical quantification of CC cell AKH peptide content (Braco et al., 2012; Kim and Neufeld, 2015; Oh et al., 2019; Hughson et al., 2021; Hughson, in press), *in vivo* cell imaging assays (Kim and Rulifson, 2004; Braco et al., 2012), pharmacology (Kim and Rulifson, 2004), and changes in systemic lipid and carbohydrate metabolism (Kim and Rulifson, 2004; Lee and Park, 2004; Isabel et al., 2005; Grönke et al., 2007; Hughson et al., 2021; Hughson, in press).

The precise quantification of circulating sugar (i.e., glucose and trehalose) titers is essential for modeling metabolic syndrome in *D. melanogaster*. Existing assays are efficient and highly replicable, and use enzymatic reactions that permit colorimetric sample quantification (Buch et al., 2008; Musselman et al., 2011; Tennessen et al., 2014; Kim and Neufeld, 2015). The ideal protocol will also allow for quantification of lipid and hormone (e.g., AKH, DILPs, ecdysteroids) titers from the same hemolymph sample. High performance liquid chromatography (HPLC) has been used to quantify glandular and hemolymph ecdysteroid titers (Yamanaka et al., 2015). Mass spectrometry (MS) methods benefit from high sensitivity and requirement for low sample volumes and can be used in conjunction with isotope labeled nutrients and hormones. Combined HPLC and MS techniques were used to quantify tissue specific lipid accumulation (Tuthill et al., 2020). Liquid chromatography tandem MS (LC-MS/MS) experiments can detect and quantify ^13^C-labeled glucose in hemolymph drawn from flies reared on the labeled medium (Cocuron et al., 2020). AKH titers can be quantified by spiking samples with a known quantity of isotope-labeled AKH (e.g., Sigma AQUA peptides). Another MS technique, tandem mass tagging (TMT) of proteins and nucleic acids, permits sample multiplexing.

These HPLC and MS methods require hemolymph sampling, which in larvae is a simple process of tearing the cuticle and allowing hemolymph to pool on/in a glass slide, capillary, or tube (Musselman et al., 2011). In adults, the relatively small hemolymph volume and sclerotized cuticle makes sample collection more challenging than in larvae. Hemolymph can be extracted from adults by poking holes in the cuticle or removing the head and spinning the flies in a centrifuge (Tennessen et al., 2014; Gàlikovà et al., 2015). An alternative method that does not require anesthesia involves placing an adult inside a trimmed pipette tip, amputating one antenna, and apply low air pressure to the body to exude a droplet of hemolymph (MacMillan and Hughson, 2014).

#### Standardization of Lipid Content to Body Size

Another essential development for *D. melanogaster* metabolic syndrome research is a clinically relevant measure of obesity. The body mass index (BMI) standardizes body mass to body size (using height as a surrogate measure of size) and is used to diagnose overweight and obese humans (Gutin, 2018). Obesity in flies is typically reported as whole body lipid content standardized to whole body protein content under the assumption that protein content is always constant across treatments and is directly proportional to body size. Whole body macronutrient quantification is superior to the use of body mass in obesity research because the distinction between lipid and non-lipid molecules cannot be made. However, the assumption that whole body protein content is always constant and proportional to body size is rarely tested. This bears great impact on fly metabolic research because transgenic and nutritional treatments that challenge energy homeostasis will stimulate protein catabolism for energy production as starvation progresses.

When whole body protein content is altered by experimental conditions it is clearly an inappropriate surrogate measure for body size in obesity research. Furthermore, it cannot be used as a constant against which lipid content is standardized for comparison between treatment groups. Alternatives to this method are to use wing surface area or thorax length as a measure for adult body size (Delcour and Lints, 1966; McBrayer et al., 2007; Gàlikovà et al., 2015). Wing/thorax measurements can be used in conjunction with whole body lipid quantification to create a Lipid Mass Index (LMI). As with the BMI, this can be calculated using the formula LMI = [lipid mass]/[wing surface area or thorax length]^2^ and yield a clinically relevant measure of obesity in flies. It needs to be noted that wing surface area is sometimes inconsistent with body size. Wing measurements may be less appropriate than thorax length, particularly where wing imaginal disk growth–mediated by DILP2 and DILP8–may be affected differentially between experimental and control lines (Brogiolo et al., 2001; Colombani et al., 2012).

Variation in genetic background can influence development and body size. The contribution of genetic background to this and other traits can be controlled through the backcrossing of mutant lines into an isogenic background (Greenspan, 2004). For life stage-specific experiments, GeneSwitch (GS) provides temporal control over transgene expression via drug (RU486, mifepristone)-dependent activation of GAL4 activity (Osterwalder et al., 2001; Roman et al., 2001). This controls for the effects of genetic background mutations on development in transgenic experiments, and recent advances have helped to reduce RU486 side effects (Robles-Murguia et al., 2019). In adult life stage-specific research, GS-GAL4 prevents developmental effects of GAL4-UAS activity in the experimental F1 line that cannot be controlled for in the GAL4 and UAS control lines (e.g., the GAL4-UAS manipulation alters DILP8 activity in imaginal wing disks in a manner not present in the GAL4 and UAS controls). The use of other GAL4 regulators (e.g., GAL80) can also provide this temporal control of GAL4-UAS activity that might alter DILP8 activity in wing disks (McGuire et al., 2003).

Genetic effects on whole body protein content that are not reflected in wing/thorax size can bias the characterization of obesity. For example, in a GAL4-UAS transgenic manipulation where there is no significant difference in lipid content and wing/thorax size between the GAL4-UAS treatment genotype and the GAL4 and UAS control genotypes but there is a significant difference in protein content in GAL4-UAS vs. the control genotypes, this will create a bias where the GAL4-UAS genotype appears more/less obese than controls. In this example, lipid content and wing/thorax size are not significantly different between the three genotypes and there is no effect of the GAL4-UAS treatment on obesity; in contrast, when the GAL4-UAS has significantly reduced protein content, lipid:protein standardization gives the false appearance of obesity in the GAL4-UAS treatment group. This kind of error is misleading and creates flawed hypotheses of obesity mechanisms.

#### Flies Are What They Eat

The international *D. melanogaster* community collaborates to study metabolic syndrome by sharing reagents and expertise. The utility and replicability of this research depends upon rearing flies in a consistent nutritional environment. While this is easily accomplished within one lab, it is rare that multiple lab groups use the same nutrient medium. Given the significant influence of nutritional history on fly development and metabolic health, the establishment of a standardized nutrient medium will aid international collaborative efforts by removing the uncertainty associated with nutrient experience when comparing experimental results between groups.

The recipe for a standardized diet was developed to meet this need in the *D. melanogaster* research community (Piper et al., 2014). This holidic diet is a precise blend of chemically defined ingredients that are available from chemical supply companies. The purity of these ingredients makes it possible for different labs to follow the same recipe and create identical nutrient media. Another benefit of using this diet is that precise nutritional manipulations are easily designed and replicated. Disadvantages of this diet include the increased cost of ingredients and time/complexity of cooking the food. There are also concerns regarding the viability of some fly lines–particularly sensitive ones–on this nutrient medium.

Researchers take great care to control the genetic background of their fly stocks to prevent confounding effects of genetic variation on their phenotypes of interest. The control of nutrient background is far simpler and prevents confounding effects of variation in nutritional history. Future efforts that modify the holidic diet–or develop new diets–provide the means to control this variable by standardizing the use of one standard nutrient medium in *Drosophila* research labs.

### Developing Areas in Adipokinetic Hormone Research

*Drosophila* has not lived up to its potential as a model organism for metabolic research (Owusu-Ansah and Perrimon, 2014). As was made clear in this review, knowledge of regulatory mechanisms governing AKH secretion lags behind that of glucagon secretion in mammals. This is one of the most important advances in AKH research that must be made to improve the utility of fly metabolism research in clinical research.

The dysregulation of intrinsic (intracellular regulation of glucagon/insulin biosynthesis and secretion) and extrinsic (external neurohormonal input that regulates biosynthesis and secretion) mechanisms of glucagon/insulin physiology contribute to the pathogenesis of diabetes (Alfa and Kim, 2016). Primary (the glucagon/insulin signaling pathways) and secondary (ligand-receptor binding and downstream signaling effectors) mechanisms in the cellular targets of glucagon and insulin signaling also contribute to pathogenesis (Alfa and Kim, 2016). Knowledge gaps in any of these four mechanisms governing glucagon and insulin physiology will severely limit the development of diabetes models. This review identified promising areas for investigations into intrinsic mechanisms of AKH physiology that will contribute to models for the pre-diabetic and diabetic states of hyperglucagonemia and hyperglycemia.

The circadian regulation of AKH biosynthesis and/or secretion is a putative extrinsic factor that should be investigated. Circadian regulation of behavior and physiology provides essential input to homeostatic control of metabolism. Energy expenditure changes between sleep and wake cycles and this requires changes in AKH and DILP secretion. The PDF is a neuropeptide that is required for maintaining circadian rhythms and activity (Renn et al., 1999; Hyun et al., 2005). Its receptor, PDFR, was identified in the CC where its activity decreased starvation resistance and increased locomotion in fed flies (Braco et al., 2020). A possible explanation for these results is that PDF signaling in the CC promoted AKH secretion.

The presence of PDFR in the CC identifies PDF signaling as a putative extrinsic factor that regulates AKH physiology. PDF signaling in the CC increased intracellular cAMP levels, an event that was reported to promote extracellular Ca^2+^ entry into the CC for Ca^2+^-dependent AKH secretion (Pannabecker and Orchard, 1987; Sabado et al., 2017; Braco et al., 2020). cAMP promotes voltage gated ion channel conductance in excitable cells and is known to directly phosphorylate cardiac L-type VGCCs (Siggins, 1982; Gao et al., 1997). It is possible that PDF acts in the CC to modulate cell membrane excitability by promoting cAMP phosphorylation of a VGCC subunit (Perry et al., 2020). In this putative role, PDF confers the regulatory influence of circadian rhythmicity upon AKH secretion.

An exciting area for future research lies in characterizing the functional parallels between the AKH and GnRH orthologs. Activation of the HPG axis through GnRH signaling at the onset of puberty stimulates the transition from juvenile to adult life in mammals (Parent et al., 2003). Juvenile metabolic stress caused by famine or low socioeconomic status perturbs HPG activity and thereby contributes to the pathogenesis of metabolic syndrome both within and across generations (Habtu et al., 1999; Painter et al., 2008; Jang et al., 2013; Wang et al., 2017).

Adipokinetic hormone altered the timing of larval development in responsive to low nutrient stress, and AKHR was implicated in the intergenerational transmission of the effect of nutrient stress on lipid homeostasis (Palu et al., 2017; Hughson et al., 2021). AKHR signaling in the fat body activated the PKA-LKB1-SIK3-HDAC4 pathway (Choi et al., 2015; Heier et al., 2021). Chronically elevated glucagon signaling suppressed SIK3 via the PKA-LKB1 pathway and caused HDAC4-mediated activation of FOXO to produce a pre-diabetic hyperglycemic state (Luong et al., 2006). This concurs with a recent report that AKHR signaling in the fat body mediated the hyperglycemic response to a high sugar diet (Song et al., 2017).

Epigenetic mechanisms play a causal role in the inheritance of acquired metabolic traits (Somer and Thummel, 2014). As an epigenetic modifier, the histone deacetylating activity of HDAC4 is a candidate mediator of intergenerational transmission of nutrient stress effects via epigenetic inheritance. The epigenetic effects of HDAC4 are particularly relevant due to its effect on the expression of genes that regulate the glycemic index (Kasinska et al., 2016). The dual functionality of AKH as a glucagon-like and a GnRH-like peptide presents great potential for understanding the etiological basis of metabolic syndrome, as well as the means whereby the effects of nutrient stress are transmitted across generations through altered HPG axis activity.

## Author Contributions

BNH confirms being the sole contributor of this work and has approved it for publication.

## Conflict of Interest

The author declares that the research was conducted in the absence of any commercial or financial relationships that could be construed as a potential conflict of interest.

## Publisher’s Note

All claims expressed in this article are solely those of the authors and do not necessarily represent those of their affiliated organizations, or those of the publisher, the editors and the reviewers. Any product that may be evaluated in this article, or claim that may be made by its manufacturer, is not guaranteed or endorsed by the publisher.
